# Probiotics in patients with severe acute pancreatitis

**DOI:** 10.1186/cc13968

**Published:** 2014-07-03

**Authors:** Wan-Jie Gu, Jing-Chen Liu

**Affiliations:** 1 Department of Anaesthesiology, the First Affiliated Hospital, Guangxi Medical University, Nanning 530021 Guangxi, China

## 

Gou and colleagues have conducted a systematic review and meta-analysis of randomized controlled trials to determine the effects of probiotics in the treatment of severe acute pancreatitis [1]. We congratulate and applaud their important work, but several important issues should be noted.

First, in the 'Methods' subsection of the Abstract, the authors stated 'Eligible studies were randomized controlled trials that compared the effects of probiotics administration with placebo treatment in patients with predicted SAP [severe acute pancreatitis]'. However, in Table two, the information for the Control group suggests that most of the eligible studies compared probiotics with control (that is, heat-inactivated *L. plantarum* 299 per serving, bioactive fibers, or water) rather than placebo. The two parts seem inconsistent.

Second, in the 'Inclusion and exclusion criteria' subsection, the authors stated 'We included all human RCTs [randomized controlled trials] that investigated the effects of probiotics in SAP patients', but the trial of Plaudis and colleagues [2] was just a prospective feasibility study. Whether this study was a randomized controlled trial or not remains unclear, just as acknowledged by the authors in Table one. Strictly speaking, the Plaudis and colleagues trial should not be included.

Third, in the 'Statistical analysis' subsection, the authors stated 'The Mantel-Haenszel random-effects model and fixed-effects model were used for data with and without significant heterogeneity, respectively'. What is the meaning of significant heterogeneity? I^2^ > 50% or something else? It is ambiguous and the authors need to clarify.

Lastly, in Figure two, Besselink and colleagues [3] accounts for 69.9% (pancreatic infection), 81.2% (total infection), and 84.1% (mortality) of the total weight. The authors should perform sensitivity analysis by omitting the Besselink and colleagues trial to test the robustness of their results.

## Competing interests

The authors declare that they have no competing interests.

## Authors’ contributions

WJG and JCL conceived the study, participated in the design, collected the data, and drafted the manuscript. All authors read and approved the final manuscript.
